# Functional and Phylogenetic Diversity of BSH and PVA Enzymes

**DOI:** 10.3390/microorganisms9040732

**Published:** 2021-03-31

**Authors:** Jack W. Daly, Stephen J. Keely, Cormac G. M. Gahan

**Affiliations:** 1 Alimentary Pharmabiotic Centre Microbiome Ireland, University College Cork, T12 YT20 Cork, Ireland; jack.daly@ucc.ie; 2 School of Microbiology, University College Cork, T12 YN60 Cork, Ireland; 3 Department of Molecular Medicine, RCSI Education and Research Centre, Beaumont Hospital, D09 V2N0 Dublin, Ireland; skeely@rcsi.ie; 4 School of Pharmacy, University College Cork, T12 YN60 Cork, Ireland

**Keywords:** bile salt hydrolase, penicillin V acylase, cholesterol, bile acid, microbiome, FXR, TGR5

## Abstract

Bile salt hydrolase (BSH) and penicillin V acylase (PVA) are related enzymes that are classified as choloylglycine hydrolases (CGH). BSH enzymes have attracted significant interest for their ability to modulate the composition of the bile acid pool, alter bile acid signaling events mediated by the host bile acid receptors FXR and TGR5 and influence cholesterol homeostasis in the host, while PVA enzymes have been widely utilised in an industrial capacity in the production of semi-synthetic antibiotics. The similarities between BSH and PVA enzymes suggest common evolution of these enzymes and shared mechanisms for substrate binding and catalysis. Here, we compare BSH and PVA through analysis of the distribution, phylogeny and biochemistry of these microbial enzymes. The development of new annotation approaches based upon functional enzyme analyses and the potential implications of BSH enzymes for host health are discussed.

## 1. Introduction

**Figure 1:**
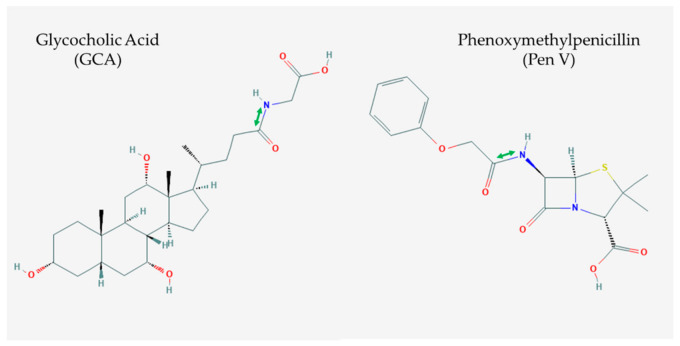
The 2-dimensional chemical structure of glycocholic acid (GCA) (PubChem identifier: 10140) and phenoxymethylpenicillin (Pen V) (PubChem identifier: 6869). The amide bonds hydrolysed by either bile salt hydrolase (BSH) or penicillin V acylase (PVA) are annotated by the green arrow.

Bile salt hydrolase (BSH) and penicillin V acylase (PVA) are related enzymes that are classified as choloylglycine hydrolases (CGH) within the Ntn (*N*-terminal nucleophile) hydrolase enzyme superfamily [1]. This superfamily is characterised by the catalytic mechanism which involves a nucleophilic attack by the *N*-terminal residue on the substrate carbonyl carbon of the amide bond [2,3]. The Ntn-hydrolase superfamily possesses a distinctive four-layered catalytically active αββα-core structural fold [2]. The core consists of two antiparallel β sheets packed against each other and covered by a layer of antiparallel α helices on one side [4]. The Ntn-hydrolase fold permits a broad range of substrate specificities between enzymes with only minimal modifications to the active site and substrate binding pocket [5]. BSH and PVA enzymes share many common structural features and are proposed to be evolutionarily related. The most significant distinguishing characteristic is their substrate specificity, with BSH targeting conjugated bile acids and PVA hydrolysing phenoxymethylpenicillin (penV) (Figure 1) [6]. Previous studies have observed distinct phylogenetic clustering of CGH proteins identified from either Gram-positive or -negative bacteria [7,8,9,10]. While PVA enzymes are predominantly found in soil and aquatic microbes, BSH enzymes are widely abundant among microbes in the gut [10]. Functional metagenomic analysis by Jones et al. (2008) demonstrated that active BSH proteins were typically found in gut-related bacterial communities, whereas genes encoding PVA were also found in marine and soil metagenomes. This is corroborated by a recent study by O’Flaherty et al. [9] in which BSH encoding genes were typically found in gut-related *Lactobacillus* species, while genes encoding PVA were associated with *Lactobacillus* species from a wider variety of environments. These findings highlight a potential niche-specific evolutionary adaptation associated with BSH enzymes, towards gut-specific functionality that may benefit both bacterium and host. Due to the importance of PVA and BSH enzymes in terms of both health and industrial applications, accurate classification and differentiation are imperative to select enzymes with optimal activities for specific uses. The following sections will examine the structural similarities and differences between PVA and BSH enzymes and methods used for annotation of genes encoding putative enzymes.

## 2. An Overview of Enzyme Function and Distribution

### 2.1. BSH Function and Distribution

Bile acids are water-soluble products of cholesterol synthesised in the liver [11,12]. In the small intestinal lumen they act as detergent molecules, facilitating the breakdown and absorption of dietary lipids through emulsifying activity [13]. They also act as signalling molecules in the host through activation, and inhibition, of specific bile acid receptors, including the farnesoid X receptor (FXR) and the Takeda G-protein-coupled bile acid receptor (TGR5) [11,14]. Primary bile acids, cholic acid (CA) and chenodeoxycholic acid (CDCA), are amphipathic molecules synthesised through either the classical or alternative pathways in hepatocytes [14]. These primary bile acids are subsequently conjugated to the amino acids glycine and taurine to form bile salts which are stored in the gallbladder [11]. The ratio of glycine-based conjugated bile salts to their taurine counterparts in humans is approximately 3:1 [15].

**Figure 2:**
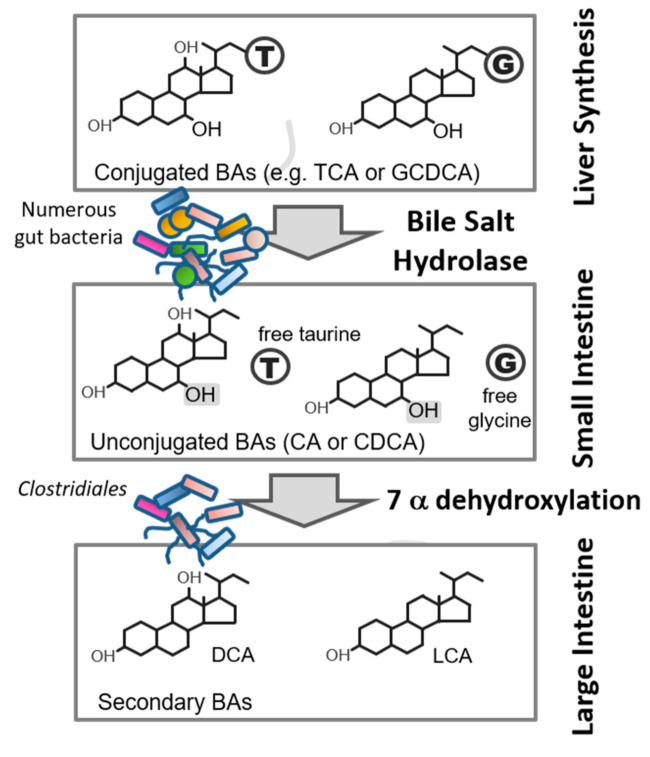
Microbial metabolism of bile acids. Primary bile acids (BAs) are conjugated to an amino acid, either glycine (indicated by “G”) or taurine (indicated by “T”), in the liver to form conjugated bile acids, such as taurocholic acid (TCA) and glycochenodeoxycholic acid (GCDCA). In the intestinal tract, numerous gut bacteria exhibit bile salt hydrolase enzymatic activity capable of cleaving the amide bond of conjugated bile acids resulting in unconjugated primary bile acids and freed glycine or taurine. Certain gut bacteria may act upon these unconjugated primary bile acids through further enzymatic activity, such as 7α-dehydroxylation, to form secondary bile acids, such as deoxycholic acid (DCA) and lithocholic acid (LCA).

Microbiota inhabiting the ileum and colon deconjugate bile salts through their BSH activity. This enzymatic activity hydrolyses the amide bond, freeing the bile acids from their associated amino acids (Figure 2) [16]. The BSH enzyme has been found amongst all known major phyla of the gut microbiome, including the Firmicutes, Bacteroidetes, Actinobacteria and Proteobacteria [7,11]. Furthermore, potentially active enzymes have been observed across two domains of life within the gut microbiome, bacteria and archaea [7]. While this study observed BSH enzymes to be widely dispersed within gut bacteria and archaea, it also highlighted subtle differences in the BSH proteins observed in human and murine gut environments potentially reflecting differences in bile acid pool composition and highlighting host species specificity of the enzyme [7].

The widespread prevalence of BSH has resulted in the evolution of multiple isoforms of the enzyme and several studies have proposed classification mechanisms to aid in future identification and characterisation of BSH enzymes. Phylogenetic analyses have previously subdivided the enzymes into sub-groups of four [17], five [18], eight [19] and nine [7] isoforms with another study, implementing sequence similarity networks, creating seven groups of classification [20]. Interestingly, several bacteria possess multiple BSH homologues which may exhibit a wide variation in catalytic capacity and substrate specificity [1,19]. Previous studies have classified some of these homologues into different phylogenetic clusters. An investigation into the prevalence of BSHs based on data from the human microbiome project identified 591 BSHs spread across just 447 strains as a result of several strains possessing multiple BSH alleles [19]. The study observed that 72.84%, 23.49%, 3.36% and 0.67% of BSH expressing strains possessed encoded one, two, three and four BSH paralogues, respectively. Minimal sequence similarity between multiple BSHs and the flanking regions coupled with the presence of proteins that are often inserted in or associated with mobile genetic elements suggest that these multiple genes may have been acquired through horizontal gene transfer [1,21,22,23]. The organisation and location of these genes appear to vary considerably. The BSH genes of *Lactiplantibacillus plantarum* WCFS1, *Lactobacillus acidophilus* NCFM, *L. gasseri* ATCC 33323, and *L. gasseri* V-V03 are expressed as monocistronic transcripts [9,24] whereas *L. johnsonii* 100-100 appears to possess an operon of functionally related BSH genes [21]. An investigation into the genomic location of these genes was performed on *Lactobacillus* species containing multiple BSH copies [18]. Various BSH types within one strain are not generally clustered. However, the location of the same BSH gene in multiple strains of the same species are relatively fixed.

It is thought that bacterial BSH activity may have evolved to allow microbes to survive the antimicrobial effects of bile acids. Observations suggest that the integrity of the bacterial membrane can be compromised by bile acids, with conjugated bile acids exhibiting more deleterious effects relative to unconjugated bile acids [6,25]. In addition, regulation of intracellular pH within bacterial cells may be facilitated by BSH activity which, in turn, prevents the harmful effects of decreased pH due to the presence of bile acids [26]. It has also been suggested that the liberated amino acid, resulting from BSH deconjugation, can be utilised as an energy source for the autochthonous bacteria of the gut. Both glycine and taurine can be metabolised as carbon and nitrogen sources for bacterial metabolism [11]. The deconjugation of these bile acids, through BSH activity, may serve as a bile tolerance mechanism allowing greater survival and colonisation [7,13]. In support of this, subcloning of isolated BSH genes into *Listeria innocua*, a strain lacking autochthonous BSH, was shown to result in an increase in bile tolerance and survival in the murine intestinal tract [7]. The prevalence of BSH amongst pathogenic bacteria, such as *Listeria monocytogenes*, suggests a role for the enzyme as a virulence factor that favours gut colonization, with BSH mutants surviving relatively poorly in the murine GI tract [26]. A recent study utilising deletion mutations of BSH in *L. acidophilus* and *L. gasseri* strains showed that, while BSH enzymes contributed to enhanced survival against specific bile acids in vitro, the enzymes were dispensable for survival in germ-free mice and in an ex vivo caecal survival model [27]. These data suggest subtleties in the requirement for BSH that may reflect bile acid specificities, the particular model systems employed, or adaptation to micro-niches in the gut.

### 2.2. PVA Enzyme Activities and Distribution

Penicillin acylases, or beta-lactam acylases, are microbial enzymes that hydrolyse the amide bond of beta-lactam antibiotics. Penicillin acylases exhibit either a substrate affinity towards benzylpenicillin, penicillin G acylases (PGA), or phenoxymethylpenicillin, PVAs [28]. Penicillin acylases have been identified across a plethora of bacterial and fungal species with PGAs being previously referred to as bacterial acylases and PVAs as fungal acylases, although it is now apparent that numerous bacterial species also express PVA [29]. Both PGA and PVA enzymes have found widespread industrial applications in the generation of the pharmaceutical intermediate 6-aminopenicillanic acid (6-APA) used in the production of semi-synthetic antibiotics [10,30]. While bearing similar catalytic features such as the αββα structural fold common to Ntn-hydrolases, PVA and PGA enzymes have low sequence homology [31,32]. PGAs possess a serine as the N-terminal catalytic residue and a heterodimeric structure whereas PVAs contain a cysteine as the catalytic residue and a homotetrameric structure [29]. The PVA enzyme, in fact, bears a closer evolutionary relationship with BSH than to PGA as exhibited by homology analyses [29,33,34]. Despite the high levels of structural similarity, sequence analysis has highlighted an evolutionary diversion between BSH and PVA proteins [34].

**Figure 3:**
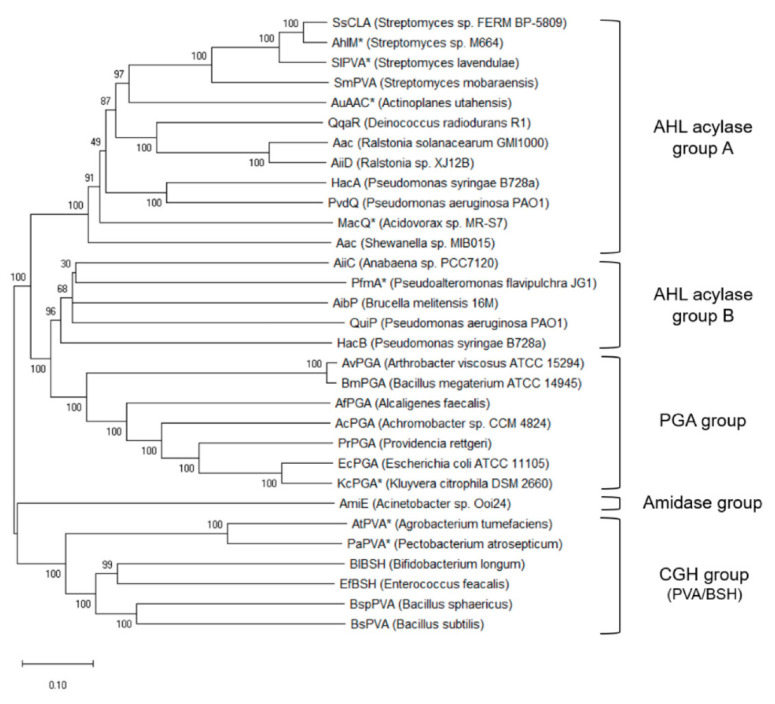
Molecular phylogenetic analysis of several members of the Ntn-hydrolase superfamily. Five clusters emerge correlating with substrate specificity: AHL acylase-like proteins are divided between two distinct clusters, *AHL acylase group A* and *AHL acylase group B*. The *PGA group* consists of penicillin G acylase proteins. The closely related bile salt hydrolase and penicillin V acylase proteins are grouped within the *CGH group*. A novel *Amidase group* was identified composed of an AHL acylase-like protein from *Acinetobacter* sp. Ooi24. Proteins that exhibit both AHL acylase and penicillin acylase activities are indicated by asterisks. The evolutionary history was inferred using the neighbour-joining (NJ) method within the 3DM and MEGA X packages. From [40].

The role of PVA in bacterial physiology and lifecycle is not fully understood. In contrast, PGA has been hypothesised to function as a scavenger enzyme involved in the catabolism of alternative carbon sources [35]. When existing within the free-living state, PGA may degrade compounds with a phenylacetyl group to utilise as a source of carbon, although this system is redundant when the organism is existing as a parasite [36]. However, this activity has not yet been observed in PVA enzymes. It is postulated that PVA may act upon other substrates in its natural environment but is often characterised primarily on its ability to hydrolyse penV, since this represents its primary industrial application [24]. Some evidence indicates that PVA may play a role in quorum quenching, thus offering a potential competitive advantage to these microbes [37,38,39]. Phylogenetic analysis has elucidated the evolutionary relationships between PVA proteins and proteins commonly associated with quorum quenching, N-Acyl homoserine lactones (AHL) acylase (Figure 3) [40]. The close evolutionary relationships between PVA and AHL acylase proteins is further compounded with examples of previously characterised PVAs exhibiting catalytic activity towards quorum sensing compounds [24,40,41,42]. These findings highlight the possible role PVA enzymes play in quorum quenching.

## 3. Biochemistry of BSH and PVA Enzymes

### 3.1. Biochemical Overview of BSH and PVA Enzymes

PVA and BSH proteins exhibit high levels of similarity, both contain the αββα Ntn-hydrolase fold which comprises highly conserved amino acids within the active centre and the ability to cleave non-protein amide bonds through the *N*-terminal nucleophilic residue, Cys [11,29,43]. The proteins are encoded by a relatively similar number of amino acids with BSH encoded by 314-338aa (Table 1) [6,17] and PVAs being, on average, slightly larger at 326-355aa (Table 1) [29]. This is likely due to insertions at the binding site of PVA [44]. Optimum hydrolytic activity for BSH proteins occurs at a pH range of 3.8–7.0 and temperatures of 30–55 °C, whereas PVA activity is optimal within a pH range 5–8 but remains stable between pH 3–10.5 and has an optimal temperature range between 40–60 °C (Table 1) [17,45,46]. It is worth noting that PVAs are stable across a broader pH range than their PGA counterparts [47]. Following the removal of the initiation formyl methionine by an autoproteolytic process, the Cys2 amino acid becomes the catalytic centre of the reaction [1]. As a consequence of their notable levels of similarity, these enzymes are annotated under a single family across public domain databases; CBAH family in Pfam, Ntn-CGH-like family in CDD; C59 family in MEROPS [10].

While both PVA and BSH proteins are generally homotetramers, certain BSHs exist as homodimeric, homohexameric and homooctameric forms while some PVAs have been observed to display a heterodimeric form (Table 1) [6,17,34,40]. Interestingly, certain BSH homologues residing in the same organism may exhibit different monomer structures [17]. The homotetrameric BSH and PVA proteins are comprised of subunits with molecular weights of 34–42 kDa and 30–35 kDa, respectively [17,29]. As previously mentioned, CGH members from Gram-negative and positive bacteria distinctly cluster through phylogenetic analysis [8,9,10]. Sequence analysis observed a 13–19 amino acid indel in Gram-negative bacteria corresponding to the absence of an assembly motif [10]. While the authors suggest this reduces the thermostability of the tetramer, a PVA from *Pectobacterium atrosepticum* was not found to dissociate into dimers in the presence of denaturing agents [55].

### 3.2. Catalytic Mechanism

**Figure 4:**
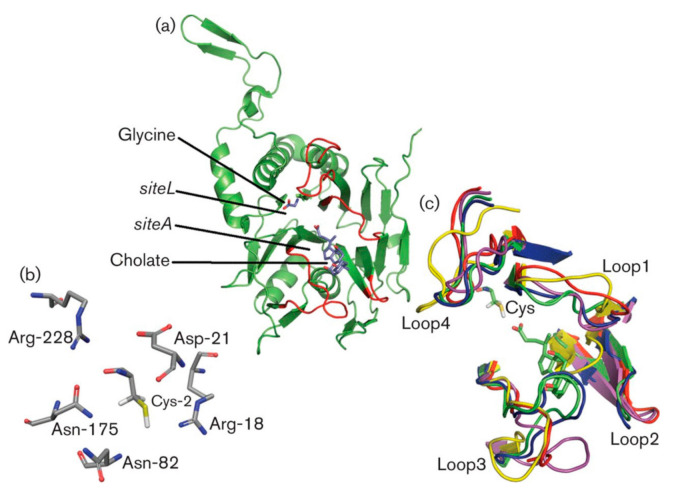
(**a**) The 3D structure of *Clostridium perfringens Cp*BSH following the hydrolysis of glycocholic acid (GCA) with the products glycine bound in the active site (siteL) and cholate bound in the binding site (siteA) (both products are shown in stick representation and labelled). (**b**) The geometrical arrangement of the six major catalytic residues in the active site of *Cp*BSH. (**c**) The superimposition of the four loops of the substrate binding site (loop1-loop4) from *Bifidobacterium longum Bl*BSH (red), *Cp*BSH (magenta), *Bacteroides thetaiotaomicron Bt*BSH (yellow), *Lyinibacillus sphaericus Bsp*PVA (blue) and *Bacillus subtilis Bsu*PVA (green). The active site nucleophilic residue Cys is shown and labelled. From [10].

As a consequence of BSH and PVA likely sharing a similar evolutionary origin, several residues involved in the catalytic mechanism are strictly conserved resulting in similar mechanisms of substrate hydrolysis. It is worth noting that protein structure is far more stable relative to the associated nucleotide sequence [56,57]. Therefore, while sequence similarity may vary considerably among CGH members, the protein structure remains relatively conserved. The active site of these enzymes lies between the two β sheets of the αββα -Ntn-hydrolase fold [19,43]. Five of the six major catalytic residues are conserved among both BSH and PVA [34,58]. The major conserved functional catalytic residues among previously crystallised BSH members are Cys2, Arg18, Asp21, Asn81, Asn175, and Arg228, numbered according to *Cp*BSH of *Clostridium perfringens* (hence, *Cp* nomenclature), the geometrical structure of which is presented in Figure 4b [19,58]. Similar conserved catalytic site residues have been observed in PVA, with the exception of an aromatic amino acid (either Phe, Tyr or Trp) which replaces Asn81 and aids in stacking interactions with the phenyl ring of penV [3,8,43,47,58].

Due to the conserved catalytic residues between BSH and PVA proteins, both exhibit highly similar hydrolytic reaction mechanisms [59]. The catalytic mechanism of CGHs comprises a nucleophilic attack by the *N*-terminal residue on the substrate carbonyl carbon of the amide bond, followed by the formation of a tetrahedral intermediate which is stabilised by an oxyanion hole [2,3]. An autocatalytic endoproteolytic process activates Cys2 (following amino acids numbered according to *Cp*BSH sequence), forming a free α-amino group serving as a base in the catalytic reaction [2,4]. The α-amino group forms a hydrogen bond with a water molecule, thereby bridging to the nucleophilic cysteinyl sulphur [6]. The negatively charged sulfhydryl group is stabilised by Arg18, which may also be involved in autocatalytic processing [3]. The *N*-terminal forms the catalytic Ntn-diad with Asp21 [3,17]. The amide bond of either conjugated bile acids or phenoxymethylpenicillin is the subject of the nucleophilic attack by Cys2 [6]. Subsequently, the tetrahedral intermediate is stabilised by the oxyanion hole formed through the NH peptide of Asn81 (Tyr/Trp/Phe in PVAs) and the N*δ*2 Asn175 in the loop [3,52]. Asn175 is involved in substrate recognition, while Arg228 aids in transition-state stabilisation [17,52]. In the case of BSH, following the release of the cleaved amino acid, a second nucleophilic attack on the thioester bond between Cys2 and the substrate liberates the cholate moiety and regenerates free enzyme through another tetrahedral intermediate [6]. As previously mentioned, PVAs possess an aromatic amino residue instead of Asn81 that aids in stacking interactions with the phenyl ring of penV [6].

### 3.3. Substrate Binding and Specificity

While the catalytic sites of both PVAs and BSHs share significant levels of similarity and a conserved reaction mechanism, subtle variations in structural elements and residues involved in substrate binding are apparent between both enzymes and likely result in a wide variety of amide substrate specificities [29,43]. The crystal structures of four BSH enzymes have previously been elucidated; *Bl*BSH from *Bifidobacterium longum* [34], *Cp*BSH from *C. perfringens* [3], *Ef*BSH from *Enterococcus faecalis* [43] and *Ls*BSH from *Lactobacillus salivarius* [50,60]. Similarly, the crystal structures of three PVA enzymes have been described; *Bsu*PVA from *Bacillus subtilis* [53], *Bsp*PVA from *Lysinibacillus sphaericus* [52] and *Pa*PVA from *Pectobacterium atrosepticum* [54]. 

Choloylglycine hydrolases share four substrate binding loops surrounding the active site, with BSH loops generally shorter to facilitate the bulky steroid nucleus in the active site [6,34]. The important residues for BSH activity are present within loop 1 (20-26), loop 2 (59-68) and loop 3 (131-42) whereas no key residues have been identified in loop 4 (263-275) [17]. These loops influence the catalytic efficiency and substrate specificity of both BSH and PVA enzymes [43]. A major structural difference between BSH and PVA enzymes lies in the length of loops in the binding pocket. Loop 3 orients itself more inside the cavity in PVA enzymes, thereby reducing the pocket size and conferring substrate specificity towards penV (Figure 4c) [29,34,43,54,55]. Through 3D structural comparisons, Loop 3 of *Pa*PVA appears to possess extra residues which are likely the cause of this inward folding by lengthening the loop [54]. Furthermore, a novel CGH identified in *Shewanella loihica* was observed to possess 4-14 more residues in loop 3 compared to *Pa*PVA and *Bl*BSH, respectively, thereby resulting in a smaller active site than in any other CGH family member reported at the time of publishing [5]. It is worth noting that an inward conformation of loop 3 was also observed in *L. salivarius Ls*BSH [61]. The loop3 region also differs between both BSH and PVA enzymes with the former exhibiting more hydrophilic polar residues and the latter more hydrophobic residues [6,55]. Furthermore, BSH enzymes are observed to have a larger and more exposed hydrophilic binding site [10]. Additionally, the side chains to loops 2 and 3 vary far more considerably in the PVA group with significant differences in size and hydrophilicity/hydrophobicity [8].

These variations in the substrate binding pocket may account for the differences in catalytic activity and specificity between these enzymes [43]. While *Bl*BSH and *Ef*BSH solely exhibit catalytic activity towards bile salts [34,43], *Cp*BSH and a BSH isolated from *L. gaserii* express moderate activity towards penV [3,56]. Conversely, *Bsu*PVA and *Pa*PVA strictly act upon penV [47,54], whereas *Bsp*PVA shows some additional catalytic activity towards bile salts [52]. A study characterising *Bl*BSH structure and function investigated *Bl*BSH, *Bsp*PVA and *Cp*BSH for catalytic activity towards both penV and bile salts [34]. While *Bl*BSH exhibited the highest BSH activity of all three, *Bsp*PVA exhibited significant activity towards both penV and taurocholic acid (TCA), albeit considerably higher towards penV. *Cp*BSH also exhibited activity towards both penV and bile salts, although the activity was marginal towards penV.

Interestingly, some BSH and PVA enzymes show non-specific binding towards both bile and penV, even though actual catalytic activity is restricted to one substrate. Through 3D molecular binding analysis, Avinash et al. [54] found that *Pa*PVA exhibited a binding affinity to both penV and glycocholic acid (GCA), with higher substrate specificity for penV. However, the enzyme failed to exhibit any functional BSH activity. This suggests that the identified enzyme, while successfully binding to GCA, does not show any catalytic activity towards the bile salt. Furthermore, a significant decrease in PVA activity was observed in the penV enzymatic assay with the addition of bile salts. A similar trend was also observed in other studies, with both *Bsu*PVA and *A. tumefaciens At*PVA activity markedly inhibited in the presence of bile salts [41,47]. Avinash et al. [54] argue that GCA binds in an inverse fashion to *Pa*PVA, hence reversing the direction of the amide bond. This is likely not ideal for substrate binding which accounts for the lack of hydrolysing activity. Notably, a study into the catalytic activity of *Ef*BSH observed a contrasting trend with the addition of penV increasing catalytic activity towards glycodeoxycholic acid (GDCA) [43]. Furthermore, the catalytic activity of both *Pa*PVA and *Bsu*PVA were significantly diminished in the presence of conjugated bile salts [47,54].

The BSH enzyme predominantly recognises potential substrates based on amino acid moieties rather than the cholate steroid nucleus [56]. Furthermore, most BSH enzymes have a greater affinity towards deconjugating glyco-conjugated bile salts than their tauro-conjugated counterparts due to the sulphur atom of tauro-conjugate bile acids causing steric hindrance [17,62]. This preference may have arisen due to the fact that glyco-conjugated bile acids are far more toxic to bacteria than tauro-conjugated bile acids, especially at low pH [6,17]. In addition, for certain host species (including humans) there is a higher concentration of glycine-conjugated bile acids relative to taurine-conjugated bile acids in the gut which may have acted as a selective pressure for this substrate preference [15]. In contrast, Ozturk et al. [58] observed considerable variation in BSH catalytic activity towards different bile acid cholate groups suggesting that BSH substrate specificity may be contingent upon the cholate group.

### 3.4. Key Residues Involved in Activity

Several studies have implemented site-directed mutagenesis and molecular docking simulations to identify key residues involved in catalytic activity. Chand et al. [43] examined whether mutating the Asn79 residue of *Ef*BSH to the aromatic residues synonymous with PVAs, Tyr and Trp, would incur catalytic activity towards penV. The mutant Asn79Tyr resulted in slightly reduced BSH catalytic activity while the Asn79Trp and a double mutant of Tyr20Trp + Asn79Trp exhibited significantly impeded BSH activity. However, none of these mutants exhibited any activity towards penV indicating that PVA activity is not solely conferred by this residue. Ozturk et al. [58] observed that a mutation of Asn79 in a *L. plantarum* BSH (*Lp*BSH) resulted in a marked decrease in BSH activity and subsequently suggested that this residue represents a major differentiating feature between PVAs and BSHs. The sequence alignment of *Ef*BSH to the previously described crystallised PVA enzymes also highlights the conserved prevalence of Met20 among PVA sequences, suggesting this may be necessary for PVA activity [43]. Interestingly, *Cp*BSH, a BSH which exhibits slight activity towards penV also possesses Met20 [34]. In contrast, a study investigating the prevalence of BSH within *Lactobacillus* species observed widespread distribution of Met20 [18].

A combination of site-directed mutagenesis and molecular docking analysis identified two aromatic residues that closely interact with the phenyl ring in *Pa*PVA, Trp23 and Trp87 [55]. It was observed that most Gram-negative PVA enzymes possess the Trp-Trp aromatic pair, whereas *Bsu*PVA and *Bsp*PVA have a Phe and Tyr aromatic pair in the corresponding positions [55]. The stability of the PVA enzyme was contingent on the strength of the aromatic interactions between the two residues while catalytic activity appeared to depend upon interactions between the aromatic residues and the penV phenyl ring. Both *Bl*BSH and *Cp*BSH possess an aromatic residue at Trp21 and Phe24, respectively, and have been demonstrated to interact with the penV molecule, although only the latter has exhibited measurable catalytic activity [3,34]. Interestingly, the replacement of Trp87 with the non-aromatic Asn, synonymous with BSH proteins, resulted in only a minimal decrease in catalytic PVA activity [55]. The Trp23 was found to be irreplaceable, however, due to its role in parallel stacking with the penV phenyl ring as evident from molecular dynamic simulations.

Xu et al. [61] utilised site-directed mutagenesis to identify the contribution of the active site residues in *Ls*BSH to reaction catalysis and substrate binding. The amino acids Tyr24 and Phe65 were mutated to validate their role in stabilising the sterane ring of the bile salts. Both resulted in a notable decrease in BSH activity towards GCA and GDCA, with mutation of Phe65 having the greatest effect, completely ablating hydrolytic activity towards GCA and GDCA and reducing activity towards glycochenodeoxycholic acid (GCDCA). This evidence suggests a likely structural role for Phe65 in sterane core stabilisation. Furthermore, BSH activity towards both TCA and taurodeoxycholic acid (TDCA) exhibited 40–50% decreases in activity relative to wild-type *Ls*BSH, while taurochenodeoxycholic acid (TCDCA) was unaffected, suggesting an alternative contribution of Phe65 to the binding of tauro-conjugated bile salts. Furthermore, Gln257 and Glu270 were analysed to identify their part in binding to the glycine moiety of bile salts. A Gln257Ala mutation was found to drastically reduce BSH activity towards glyco-conjugated bile salts, while catalytic activity towards tauro-conjugated bile salts remained unaffected, verifying the role of Gln257 in substrate recognition.

Xu et al. [30] improved the catalytic efficiency of *Bsu*PVA through directed evolution. It was observed that the mutations Ser110Cys, Asn198Tyr and Thr63Ser increased activity towards penV in the order Ser110Cys > Asn198Tyr > Thr63Ser. A 12.4-fold increase in catalytic activity was observed with all three mutations combined, thereby highlighting the roles these residues play in catalytic activity. Torres-Bacete et al. [46] examined the active sites of other penicillin acylases, identifying an additional four residues required for *S. lavendulae* to hydrolyse penV. Ser1 and His23 mutations resulted in inactive enzymes, whereas Val70 and Asn272 mutations produced enzymes with minimal activity. However, the *Sl*PVA was later recharacterized as an AHL acylase related enzyme with the authors suggesting that this is not a true PVA [40].

## 4. In Silico Differentiation and Functional Characterisation

As previously mentioned, several bacterial species possess multiple BSH homologues with each exhibiting varying degrees of catalytic efficiency and substrate preference [19]. Several *Lactobacillus* species have been shown to possess multiple BSH homologues [8,9,19,63].A study investigating the *L. plantarum* strain WCFS1 created several BSH deletion mutants to investigate the functionality of all four BSHs; designated Bsh1, Bsh2, Bsh3 and Bsh4 [24]. It was observed that Bsh1 exhibited high levels of activity, while the remaining three exhibited considerably less activity. Interestingly, Bsh3, and to a lesser extent Bsh 2 and Bsh4, exhibited activity towards penV and were observed to possess high levels of sequence similarity to other PVAs. The authors suggest that while Bsh1 is a BSH enzyme, the remaining three are likely PVA-related proteins and should be annotated as such. In contrast, a similar study investigated the role of four BSH enzymes from *L. plantarum* strain ST-III, instead using expression in *E. coli* as a means of measuring activity [64]. This study found all four BSH to exhibit significant catalytic activity towards conjugated bile salts and argued that all four putative proteins in *L. plantarum* are necessary for activity. However, the latter study did not investigate whether the BSH proteins exhibited activity towards penV. Both studies observed that Bsh1 more closely resembled canonical BSH proteins of other *Lactobacillus* species than did the Bsh2, Bsh3 and Bsh4 proteins, thereby suggesting that Bsh1 represents a true host-adapted BSH that evolved later in the evolutionary timescale [24,64].

Due to the sequence homology observed between BSH and PVA, these proteins are likely to be frequently misannotated [9]. For some isoforms, protein annotation may be based upon incomplete functional analyses in which a complete range of substrates (bile or penV) were not considered [40]. Recent studies have aimed to definitively distinguish between BSH and PVA proteins with some studies suggesting the reclassification of previously annotated proteins. Aiming to properly annotate BSH and PVA for Gram-positive bacteria, particularly *Lactobacillus*, Lambert et al. [8] implemented multiple sequence alignments, hidden Markov models (HMMs), phylogenetic profiling and 3D protein homology modelling to discern between the two proteins. The experimentally verified Bsh1 of *L. plantarum* WCFS1 was used as a seed sequence to retrieve putative CGH proteins. Phylogenetic analysis was performed on the retrieved sequences in tandem with several functionally characterised BSH and PVA enzymes. The phylogenetic analysis revealed two distinct clusters of CGH proteins, one encompassing BSH and the other PVA. Proteins that had been previously experimentally characterised fell within their respective cluster. Furthermore, proteins within the BSH cluster were derived from typical gut-related bacteria which are known to possess niche-specific BSH proteins. The study also compared the structure and capacity to bind bile salt molecules of *Bl*BSH, *Cp*BSH, *Bsp*PVA through 3D homology modelling. The differentiation of proteins and binding was similar to that of the phylogenetic analysis. The authors argue that sequences previously annotated as BSH proteins that fell within the PVA cluster have likely been misannotated and are probably PVA or PVA-related enzymes.

Panigrahi et al. [10] attempted to accurately differentiate between BSH and PVA for Gram-positive, Gram-negative and archaea using similar approaches combined with a binding site similarity-based system. Putative CGH members were retrieved using the previously characterised *Bl*BSH, *Cp*BSH, *Bsp*PVA, *Bsu*PVA, *Pa*PVA and *Bacteroides thetaiotaomicron Bt*BSH as queries. While Lambert et al. [8] found success in annotation within Gram-positive CGH members, phylogenetic profiling was found to be insufficient in terms of definitive annotation of BSH/PVA across Gram-negative bacteria and archaea. Therefore, a binding site similarity-based system was developed based on both binding sites and the mode of substrate-binding predicted through molecular docking analyses. The binding site similarity score system was determined for the 6 aforementioned characterised CGH proteins. The efficacy of the system was substantiated through the correct classification of 19 previously characterised CGH members. Similar structural differences in the 3D models of BSH and PVAs found in Lambert et al. [8] were also observed in this investigation, upholding the previous finding in which the third loop of the substrate-binding pocket orientates inwards to the cavity in PVA enzymes (Figure 4c). This study again observed BSH proteins mainly in gut-associated microbes [10].

Accuracy of annotation of BSH and PVA enzymes were also investigated in a recent similar study by O’Flaherty et al. [9]. The group built upon the work of Lambert et al. [8] and Panigrahi et al. [10] and implemented HMMs to distinguish BSH and PVA within 170 sequenced *Lactobacillus* species. A BSH reference set of sequences, consisting of 26 previously biochemically characterised BSH proteins, was implemented to screen against a database of complete and draft *Lactobacillus* sequences at NCBI. The sequences were then screened using HMMs based on the 26 sequences of the BSH reference set and a PVA reference set consisting of 8 previously biochemically characterised PVA proteins, resulting in the identification of 490 putative BSHs and 1,149 PVAs. It is worth noting that three of the reference sequences in the PVA set were Bsh2, Bsh3 and Bsh4 of *L. plantarum* WCFS1. Phylogenetic analysis corroborated the findings highlighting two distinctive separate clades consisting of either BSH or PVA. Of the 170 species, 82 species (48.24%) encoded PVA proteins, 39 species (22.94%) encoded BSH proteins, 8 species (4.71%) encoded both BSH and PVA proteins, and 57 (33.53%) species encoded neither.

## 5. Potential Role of BSH and PVA in Environmental Survival, Host Interaction and Metabolism

### 5.1. Role of BSH in Host Physiology and Metabolism

BSH activity of the gut microbiota represents the gateway reaction in the microbial metabolism of host bile resulting in the generation of unconjugated bile acids and ultimately, through the activity of other microbial enzymes, secondary bile acids (such as lithocholic acid (LCA) and deoxycholic acid (DCA) (Figure 2). Variations in levels of BSH activity therefore influence the detergent/emulsifying capacities of bile acids as well as the signalling properties of the bile acid pool. BSH activity is associated with the modulation of several aspects of host physiology such as reduction in serum cholesterol levels, regulation of dietary lipid absorption and a variety of molecular responses that are influenced by the ability of bile acids to engage with bile acid receptors, such as the farnesoid X receptor (FXR) and the Takeda G-protein-coupled receptor 5 (TGR5) [1,11,26,65]. The greater solubility of conjugated bile salts improves the formation of micelles, thus enhancing the absorption of dietary lipids in the intestine [1,26]. BSH activity has the potential to reduce the absorption of dietary lipids through the generation of unconjugated bile acids which are more hydrophobic and less soluble [1,13,66]. The decreased solubility of these bile acids also results in a reduced capacity for passive uptake by IBAT/ABAT transporters in enterocytes, thereby decreasing enterohepatic recirculation of bile acids and increasing bile acid excretion through faeces [11,67,68]. The resulting decrease in the size of the circulating pool of bile acids may be restored through increased de novo synthesis from cholesterol, thereby reducing serum cholesterol levels [6,11,67]. This mechanism is thought to underpin the cholesterol-lowering effects of BSH+ probiotic strains [69].

The activation of the FXR is associated with lipid, glucose, and energy metabolism, as well as the maintenance of triglyceride and cholesterol homeostasis [16,65,70]. Intestinal FXR activation controls bile acid synthesis in the liver via a negative feedback loop. FXR activation in enterocytes triggers the production of the enterokine fibroblast growth factor 19 (FGF19), a hormone secreted into the portal circulation, which subsequently leads to repression of the rate-limiting enzyme responsible for bile salt synthesis cholesterol 7 alpha-hydroxylase (CYP7A1), thereby inhibiting further BA synthesis [71]. Conjugated bile acids may only activate FXR in cells co-expressing plasma membrane bile acid transporters while unconjugated bile acids are free to enter cells and stimulate FXR [72]. Moreover, in vivo studies have shown that unconjugated bile acids are significantly more effective at FXR activation than conjugated bile salts [6].

Additionally, BSH activity can potentially influence the activation of TGR5 through alterations to the composition of the bile acid pool. The G-protein coupled receptor has been found to activate various intracellular signalling pathways, involving basal metabolism homeostasis and energy expenditure [73]. The secondary bile acids LCA and DCA, generated from unconjugated bile acids, have the greatest capacity for TGR5 activation, with LCA being the most potent endogenous ligand [74,75]. Activation of enteroendocrine bound TGR5 stimulates the secretion of glucagon-like peptide 1 (GLP-1), thus promoting pancreatic insulin secretion and improving insulin sensitivity [16,70]. Activation of TGR5 has consequences for energy metabolism in adipose tissue as stimulation of TGR5 in mice fed a high-fat diet can reduce weight gain and prevent insulin resistance [76]. 

The concept that bacterial BSH may influence signalling processes in the host has led to experiments to investigate the physiological consequences of BSH positive bacteria on the host. Despite the fact that unconjugated bile acids can pass freely into enterocytes to activate the FXR, a study by Degirolamo et al. [77] in mice showed that a live BSH positive probiotic cocktail generated unconjugated bile acids that did not stimulate local FXR but rather were excreted, leading to elevated de novo bile salt synthesis in the liver. A more recent study also demonstrated that a BSH expressing probiotic strain, in this case *L. plantarum* H6, altered bile acid profiles in mice on a high cholesterol diet leading to increased bile acid excretion, upregulation of de novo bile acid synthesis and a reduction in serum cholesterol [78].

Cloned BSH expressed in *E. coli* delivered to the GI tract was capable of altering systemic bile acid profiles in gnotobiotic mice and influenced a wide variety of host signalling pathways (including those involved in energy metabolism, local immunity and circadian rhythm), although a specific role for FXR was not examined [79]. In the same study colonization of conventionally raised mice with the BSH expressing *E. coli* reduced weight gain when mice were fed a high fat diet. In other studies, reduced BSH activity was associated with antibiotic treatment and correlated with increased weight gain both in humans [80] and animals [81]. In contrast, the antioxidant tempol reduced BSH activity of the microbiota and prevented obesity in mice fed a high fat diet [82,83]. The conflicting evidence with respect to weight gain may indicate differences in the models used and variations in the ability of bile acid pools to stimulate TGR5 which is associated with weight loss [76] or FXR which is associated with weight gain [82]. Studies of individual *Lactobacillus* species as probiotics in mouse models have indicated that some strains promote weight gain while others induce weight loss, with the effects not directly correlated to BSH activity [84]. It is likely that subtle variations in BSH substrate preferences may influence the effects on the murine host with BSH enzymes active against T-βMCA (a potent FXR antagonist) predicted to play the most significant role [85]. Certainly, BSH activity is important for host physiological processes and bile acid homeostasis. Indeed, a number of disease states in humans including irritable bowel syndrome (IBS), inflammatory bowel disease (IBD) and sensitivity to *Clostridium difficile* infection are correlated with reduced host BSH activity and de-regulated bile acid pools [86,87]. 

### 5.2. Role of PVA in Environmental Modulation and Survival

As previously mentioned, PVA may be involved in quorum quenching that can offer a competitive advantage during its free-living state. Quorum quenching is believed to be involved in optimising, recycling, and negating the detrimental effects of quorum sensing signals [88]. Quorum quenching may offer a competitive advantage to microbes capable of disrupting foreign signals, but only limited studies have corroborated this theory [37,39]. AHLs, a common quorum sensing autoinducer of Gram-negative proteobacteria, can be degraded by AHL acylase through hydrolysis of the amide bond between the homoserine lactone core and the acyl chain [40]. 

These bacterial AHL acylases belong to the Ntn-hydrolase superfamily [38]. Bacterial AHL acylase activity has been reported in several penicillin acylase related enzymes, including some PVA members. The previously biochemically characterised PVAs *At*PVA and *Pa*PVA have exhibited AHL degradation [41]. The three putative PVA enzymes from *L. plantarum* WCFS1 also exhibited activity towards AHL degradation [24]. A PVA from *S. lavendule* (*Sl*PVA), previously shown to exhibit significant activity towards penV, was shown to also mediate significant AHL degradation [40,46]. Subsequent phylogenetic analysis of the Ntn-hydrolase family delineated evolutionary relationships between members of AHL acylase, PGA and Choloylglycine hydrolase. The previously characterised *Sl*PVA was not a true PVA, however, but rather an AHL acylase that exhibited broad substrate specificity, as evident by the phylogenetic clustering (Figure 3) and the heterodimeric nature with a catalytic serine in place of cysteine. In contrast, there have also been cases of previously characterised AHL acylases exhibiting activity towards penicillin substrates. One such study identified an AHL acylase from *Acidovorax* sp that exhibited significant PGA activity [42]. The authors argue that enzymes with bifunctional QQ and penicillin acylase activity may be widespread. Two putative CGH enzymes identified from *Shewanella loihica (Slac1* and *Slac2)* exhibited significant activity towards AHL lactones but failed to act upon penV or bile salts, despite their significant levels of similarity to other CGH members [5]. The authors argue that to identify further AHL-active acylases, more distant homologs, such as members of the CGH family, should be investigated for activity towards AHL lactones.

## 6. Conclusions

BSH and PVA represent an interesting cluster of enzymes that most likely are related through evolution. It has been suggested that BSH evolved through host pressure on PVA enzymes as an adaptation that benefits both microbe (through improved bile acid tolerance) and host (through modulation of bile-mediated host-microbe signalling events) [7]. Both BSH and PVA enzymes have significant biomedical and chemical applications. PVA has applications in the chemical synthesis of novel penicillin derivatives. BSH plays a significant role in host-microbe interactions and a reduction in BSH activity is associated with microbiota dysbiosis and a number of disease states [86,89]. Both enzymes are prone to significant variations in structure with some isoforms exhibiting different substrate ranges and levels of activity. In order to rationally select the optimal isoforms for particular uses more information is needed in order to understand the relationship between structure and function of these enzymes. In particular, understanding the role of different isoforms of BSH in human/host health will be important to properly interrogate emerging metagenomic datasets and to choose optimised BSH enzymes (or probiotics) as therapeutics. Furthermore, thorough elucidation of the structure and function of BSH and PVA isoforms will be crucial in order to apply these enzymes in future settings such as disease prevention and industrial applications. Some of the studies outlined above have contributed significantly to discrimination between PVA and BSH enzymes through annotation models. However, some bottlenecks to annotation remain. Analyses indicate that there is a significant lack of functional data differentiating BSH and PVA enzymes and in particular examining substrate range and specific activities. In the future, an increased number of biochemically verified proteins could be used to create larger seed databases which in turn could be used to screen for putative proteins and aid in annotation. Large screens of functional activity coupled with genomic information will be crucial to hone the accuracy of these in silico differentiation methods and to improve annotation and rational selection of optimal enzymes.

## Figures and Tables

**Table 1:** Biochemical features of experimentally characterised CGH enzymes relevant to this review article. BSH activity towards bile salts is ranked in descending order. Bile salts screened include glyco- and taurocholic acid (GCA & TCA), glyco- and taurochenodeoxycholic acid (GCDCA and TCDCA) and glyco- and taurodeoxycholic acid (GDCA & TDCA).

Organism	Enzyme	pH	Temp.	Form	aa	Activity in Descending Order	Other Activity	References
*Clostridium perfringens*	*Cp*BSH	Optimum 5.8–6.4	-	Homotetramer	328	GCA, GCDCA, GDCA, TCA, TDCA	Positive Penicillin V	[48]
*Bifidobacterium longum*	*Bl*BSH	Optimum 5–7	40 °C	Homotetramer	315	GCDCA, GDCA, GCA, TCDCA, TCA, TDCA	Negative Penicillin V	[34,49]
*Lactobacillus salivarius*	*Ls*BSH	Optimum 5–6 (5.4)	41 °C	Tetramer and dimer	324/325	GCDCA, TDCA, TCDCA, GCA, TCA, GDCA	-	[50]
*Enterococcus fecalis*	*Ef*BSH	Optimum 5	50 °C	Homotetramer	324	GCA, GDCA, TDCA, TCA, GCDCA, TCDCA	-	[43]
*Lyinibacillus sphaericus*	*Bsp*PVA	Optimum 6	60 °C	Homotetramer	335	Penicillin V	Positive TCA	[51,52]
*Bacillus subtilis*	*Bsu*PVA	Active 5.5–9 Optimal 6.6–7.4	40 °C	Homotetramer	328	Penicillin V	-	[47,53]
*Pectobacterium atrosepticum*	*Pa*PVA	Active 3–6 Optimum 5	40 °C	Homotetramer	355	Penicillin V	Negative GCA and TCA	[44,54]
